# Genomic analysis of *Lactobacillus reuteri* WHH1689 reveals its probiotic properties and stress resistance

**DOI:** 10.1002/fsn3.934

**Published:** 2019-01-28

**Authors:** Lin Chen, Qing Gu, Ping Li, Su Chen, Yanjun Li

**Affiliations:** ^1^ Key Laboratory for Food Microbial Technology of Zhejiang Province College of Food Science and Biotechnology Zhejiang Gongshang University Hangzhou China; ^2^ Research and Develop Department Hangzhou Wahaha Group Co. Ltd. Hangzhou China

**Keywords:** comparative genomics, *Lactobacillus reuteri *WHH1689, probiotics, stress resistance

## Abstract

*Lactobacillus reuteri* (*L. reuteri*) WHH1689, which was isolated from Chinese traditional highland barley wine, exhibited high survival period at room temperature in drinkable probiotic yogurt. This article aimed to indicate the genes involved in probiotic function of WHH1689 and reveal potential stress resistance based on genomic analysis. Analysis of comparative genome with closely related *L. reuteri* strains identified special stress adaptation. MUMmer and ACT softwares were applied for collinear analysis, and OrthoMCL program was used for sequence alignment involved in distribution of protein cluster. We identified genes coding for carbohydrate transport and enzymes, carbon metabolism pathway, gastrointestinal tract resistance, adhesive ability, and folic acid biosynthesis, etc. Genome sequence and comparative genome analysis of *L. reuteri* WHH1689 demonstrated specific genes for genetic adaptation and stress resistance. Tolerance, adhesion, and folate test indicated the strain had multiple probiotics. *L. reuteri* WHH1689 has the potential to be a probiotic candidate in dairy foods.

## 1 INTRODUCTION

Probiotics are living microorganisms with adequate amount which have benefit for host health according to FAO/WHO (Manigandan, Mangaiyarkarasi, Hemalatha, Hemalatha, & Murali, 2015). *Lactobacillus* belongs to the most common specie of lactic acid bacteria (LAB) which are regarded as safe (Coeuret, Gueguen, & Vernoux, 2004). These strains existed widely in nature and are used as probiotic application in plant, vegetable, dairy, meat, and other food product. *Lactobacillus* showed survival ability through gastrointestinal tract and high adhesion for intestinal epithelial (Baarlen, Wells, & Kleerebezem, 2013). Probiotic LAB generally have benefit characteristic such as acid and bile salt resistance, antimicrobial activity, and adhesion ability which presented potential functionality for human health (Fata, Weber, & Mohajeri, 2018).

*Lactobacillus reuteri* is a facultative anaerobic, heterofermentative LAB which is used as autochthonous bacterium in most vertebrates and mammals and is known as improving the allergy constitution and preventing the recurrence of allergy (Shornikova, Casas, Isolauri, Mykkänen, & Vesikari, 1997; Speranza et al., 2018). It has been extensively application for relieving constipation through improving intestinal flora and adaptation to the gastrointestinal circumstance (Wegner et al., 2018).

*Lactobacillus reuteri* WHH1689 was isolated from Chinese traditional Highland barley wine in Tibetan Plateau and showed long‐term viability at room temperature (RT). This strain exhibits many probiotic properties including strong adhesion and high tolerance of acid and bile salt. It had strong antimicrobial activity against most pathogenic bacterium such as *Salmonella paratyphi*,* Escherichia coli*,* Staphylococcus aureus*, and *Shigella flexneri*. Most importantly, *L. reuteri* WHH1689 showed high survival rate for long‐term storage without postacidification in dairy. However, it was still limited to illuminate the molecular mechanism for long‐term surviving (Chen, Chen, Chen, Ren, Ge, Li, et al., 2018).

Comparative genomic analysis could reveal evolutionary process and genetic properties of various species based on genome map and sequences. The whole genome sequences of *L. reuteri* WHH1689 has been completed in our lab (Chen, Chen, Chen, Ren, Ge, Li, et al., 2018; Chen, Chen, Chen, Ren, Ge, Kang, et al., 2018). *L. reuteri* strains such as DSM20016 (Susan, Iyappan, Vijaya, & Rajnish, 2017), JCM 1112 (Morita et al., 2008), SD2112 (Báth, Roos, Wall, & Jonsson, 2005), I5007 (Hou et al., 2014), TD1 (Leonard et al., 2014) could be used to annotate the genome for further research. This study aimed to reveal potential genes which were responsible for their probiotic potential and genetic resistance.

## 2 MATERIALS AND METHODS

### 2.1 Bacterial strain and cell line

The whole genome sequence of *L. reuteri* WHH1689 have been previously reported and deposited at Gen Bank with accession number CP027805 (Chen, Chen, Chen, Ren, Ge, Kang, et al., 2018). The genomic sequences of other four *L. reuteri* strains are available from NCBI data base (http://www.ncbi.nlm.nih.gov/): *L. reuteri* DSM 20016 (NC_009513.1) *L. reuteri* TD1 (NC_021872.1), *L. reuteri* SD2112 (:NC_015697.1), *L. reuteri* I5007 (:NC_021494.1). *L. reuteri* WHH1689 was isolated from traditional Chinese highland barley wine. *Lactobacillus rhamnosus* GG (LGG) were obtained from CGMCC, (China General Microbiological Culture Collection Center, China). HT‐29 cell line was used for adhesion test. This human colon adenocarcinoma cell was purchased from Chinese Academy of Sciences. *Lactobacillus plantarum* LZ227 stored in our lab was used as a microbiological indicator for folic acid.

### 2.2 Gene annotation and phylogenetic tree

Whole genome sequence of *L. reuteri* WHH1689 was predicted by Glimmer 3.02 (Delcher, Bratke, Powers, & Salzberg, 2007). With regard to the functional annotation, Cluster of Orthologous Groups (COG) and Gene Ontology (GO) were adopted (Bose, Haque, Reddy, & Mande, 2015; Langille & Brinkman, 2009). For biological pathways, BLAST algorithm was used to compare the obtained predictive genes with Kyoto Encyclopedia of Genes and Genomes (KEGG) database (Altermann & Klaenhammer, 2005; Benesty, Huang, & Chen, 2003).

Based on the results of homologous gene analysis, single copy was selected for multi‐sequence comparison and quality control using MAFFT software (Katoh & Toh, 2008). Then the phylogenetic tree was constructed with the RAxML software (Stamatakis, 2006).

### 2.3 Bioinformatic analyses

OrthoMCL v2.0.3 software was used to compare the amino acid or nucleotide sequences of all the species involved in the analysis (Li, Stoeckert, & Roos, 2003). The threshold value was selected for similarity clustering to obtain gene homology (Fischer et al., 2011). The distribution of species in each proteome cluster can be counted to conduct genomic analysis within the core genomes of genus or species. Co‐linear analysis of more genome sequences was performed using MUMmer 3.0 or ACT software (Hu et al., 2006; Toropov, Vakhitov, Shalaeva, Roshchina, & Sitkin, 2018).

### 2.4 Tolerance test

Tolerance test was examined by pH, bile salt, and osmotic pressure. *L. reuteri* WHH1689 was cultured in MRS at 37°C for 18 hr under aerobic condition. The bacterial cells were collected by centrifugation (10,000 × *g* for 10 min) and washed twice with 0.01 M PBS (pH 7.2) before being resuspended in 0.85% sterile saline and adjusted using NaOH (0.5 M) or HCl (0.5 M) to different pH values (2.0, 3.0, 4.0, 5.0, 6.0, 7.0, 8.0, 9.0, and 10.0). To evaluate resistance to bile salt, bacterial cells prepared as above were resuspended in different bile salt solution containing 0.2%, 0.3%, 0.4%, 0.5% (wt/vol) bile salt (Sigma). Bacterial suspensions were cultured at 37°C for 3 hr. Sodium chloride has strong water‐reducing activity could effect on osmotic pressure. For resistance to osmotic stress, bacterial culture was collected as above and resuspended in 6.0%, 7.0%, 8.0% sodium chloride solution. Bacterial cells were cultured at 37°C for 24 hr. Stress resistance was assessed by bacterial survival.

### 2.5 Effect of high temperature on *Lactobacillus* strains

*Lactobacillus reuteri* WHH1689 and *L. rhamnosus* LGG were independently propagated and inoculated 2 ml into 200 ml of MRS. Bacteria suspension were, respectively, cultured at 45 and 50°C for 0–30 days. Effect of high temperature on *Lactobacillus* strains were determined by bacterial counts.

### 2.6 In vitro antioxidant activity assay

#### 2.6.1 Scavenging of hydroxyl radical

The ability of the hydroxyl radical scavenging assay was determined using a Fenton reaction method (Rao, Giri, Goud, & Golder, 2016). *L. reuteri* WHH1689 and *L. rhamnosus* LGG were prepared at concentration ranging from 10^7^ to 10^9^ CFU/ml. Intracellular extracts were obtained by ultrasonic broken in ice bath. The reaction mixture containing 1.5 ml bright green reagent (0.5 mM), 1.0 ml FeSO4 (1.0 mM), 1.0 ml H_2_O_2_ (2.5%, w/v) and 0.5 ml of bacteria extract in different concentration was immediately mixed and cultured at room temperature for 30 min. The scavenging ability for hydroxyl radical of stains was measured by the absorbance at 625 nm.

$$\text{Hydroxyl radical scavenging rate}\;\left(\%\right)=\frac{A_{i}-A_{0}}{A-A_{0}}\;\times \;100$$

A_*i*_ shown the absorbance of the sample, A_0_ illustrated absorbance of the control without sample, and A represented the absorbance without Fenton reaction system and e sample.

#### 2.6.2 Scavenging of DPPH radical

The DPPH scavenging assay was investigated according to the method (Lin & Chang, 2000). Intracellular extracts of *Lactobacillus* strains were collected as above. The reaction mixture was added 2 ml DPPH free radical ethanol solution (0.1 mM) and intracellular extracts of different concentration. Then the solution were quickly mixed and reacted at room temperature in the dark for 60 min. The supernatant was collected by centrifugation (10,000 × *g* for 10 min) and measured for absorbance at 517 nm. Phosphate buffer (PBS) was used as a blank control.

$$\text{DPPH scavenging rate}\;\left(\%\right)=\left(1-\frac{A_{i}}{A_{0}}\right)\;\times \;100$$

A_*i*_ demonstrated the absorbance of the sample, A_0_ shown the absorbance of the control.

### 2.7 Adherence assay

Adhesion of the strains was assayed according to the reported method (Kim, Oh, Park, & Kim, 2009). HT‐29 cell was prepared in Dulbecco's Modified Eagle Medium (DMEM) supplemented with 10% fetal bovine serum in 24‐well tissue culture plates at 1.0 × 10^6^ cells/well concentration. *L. reuteri* WHH1689 and *L. rhamnosus* LGG at concentration ranging from 10^6^ to 10^9^ CFU/ml were added into cell solution. The plates were incubated at 37°C for 2 hr under aerobic condition (5% CO_2_/95% air atmosphere). The monolayer was washed three times with sterile PBS. For detaching, 0.05% Triton X‐100 diluted in sterile solution which was used for pipetting adherent bacteria. *L. rhamnosus* LGG was control stain and every assay was performed in three times.

### 2.8 Folic acid assay

The ability of folate produced by strains was determined by folic acid assay medium. *L. plantarum* LZ227 and *L. reuteri* WHH1689 were propagated in MRS broth at 37°C for 18 hr. The bacterial cells were collected by centrifugation (10,000 × *g* for 10 min) and washed twice with 0.01 M PBS (pH 7.2). A 0.5% inoculum of culture was, respectively, distributed into folic acid assay medium at 37°C in dark for 24 hr. The folate content of cell supernatant and intracellular extracts was assayed by Vita Fast^®^ Folic acid (IFP, R‐Biopharm, Germany).

## 3 RESULTS AND DISCUSSION

### 3.1 Genome features of *L. reuteri* WHH1689

**Figure 1:**
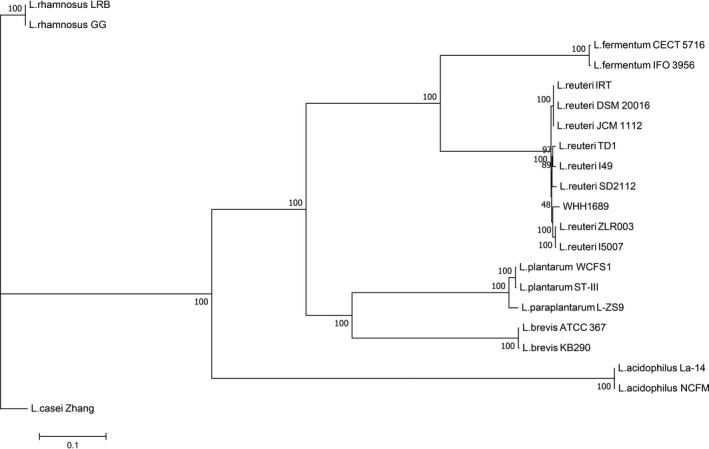
Phylogenetic tree of *L. reuteri* WHH1689 constructed from *Lactobacillus* strains based on MAFFT

**Figure 2:**
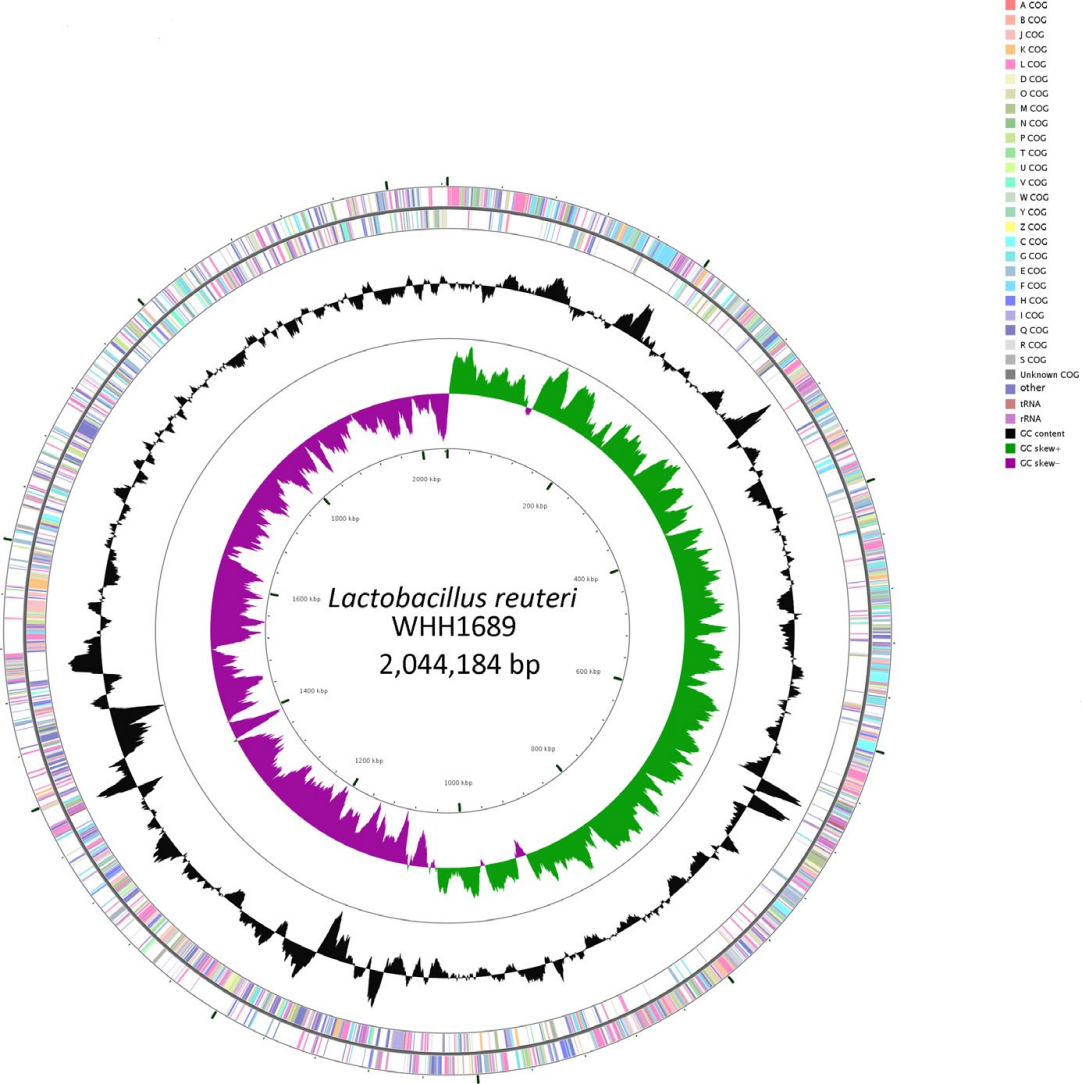
Circular genome map of *L. reuteri* WHH1689. From the inner circle: the first circle presents the GC skew (G + C/G−C), values >0 in green and values <0 in purple. The second circle depicts the GC content. The third circle depicts CRISPR repeats in black. The fourth circle highlights rRNA and tRNA, the color related to COG functional classification. The fifth to seventh circles denotes the sites of CDS

**Figure 3:**
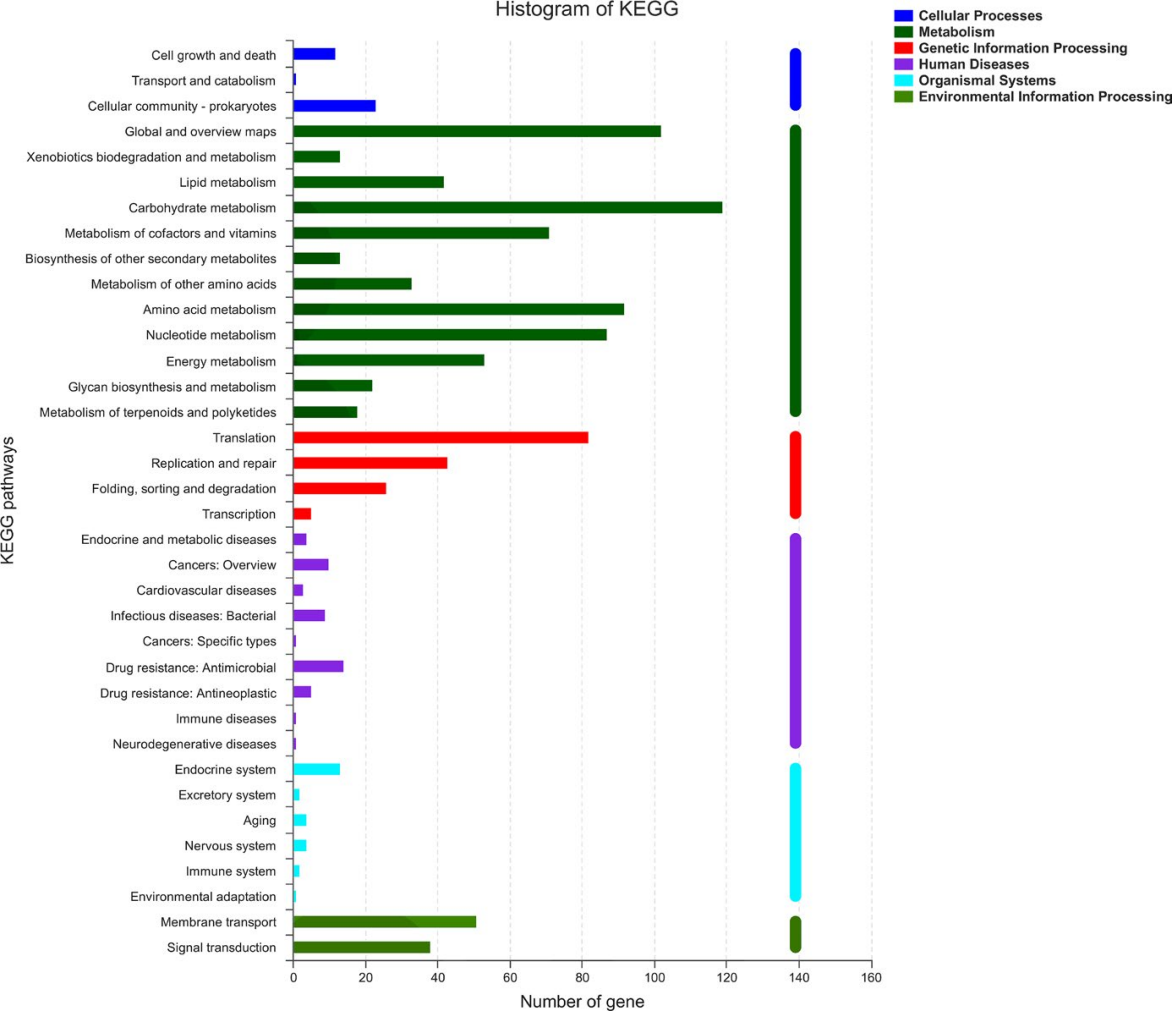
Histogram of KEGG. Histogram presents the number of genes involved in KEGG pathway. The colors indicates different systems, mazarine refers to cellular processes, bottle green depicted metabolism, red illustrates genetic information processing, purple denotes human diseases, blue highlights organismal system, and reseda shows environmental information processing

The circular genome of WHH1689 is comprised of 2,196 genes which were predicted with the average length of 814 bp. GC content accounts for 34.5% in inter genetic region. A total of 24 genomic islands involved in hundreds of functional genes including transporter, membrane protein, some enzyme, heavy metal resistance, and putative protein gene (Liu et al., 2015; Yoo et al., 2017). A phylogenetic tree of WHH1689 revealed the genetic evolution between different *L. reuteri* strains (Figure 1). It showed that different branch with *L. reuteri* strains. *L. reuteri* ZLR003 and I5007 formed the closest genetic relation with WHH1689. The circular genome map of *L. reuteri* WHH1689 was showed the genome distribution (Figure 2). The protein coding genes of WHH1689 were predicted by KEGG annotation and functionally categorized (Figure 3). In the live organism, gene products do not exist in isolation. Different gene products perform specific biological functions together through orderly coordination. KEGG annotation have abundant access information could achieve function message, such as metabolic pathway, genetic information transmission, and cytological process (Avrani, Wurtzel, Sharon, Sorek, & Lindell, 2012; Jia et al., 2017). For WHH1689, metabolism had a large proportion in histogram of KEGG. The genes involved in carbohydrate metabolism (9.78%) were most abundant, followed by amino acid metabolism (7.64%), translation (6.73%), and membrane transport (4.27%). These suggest that WHH1689 exhibited multiple metabolic pathways and was able to adapt environmental conditions (Crowley, Bottacini, Mahony, & Van, 2013; Wegmann et al., 2009).

### 3.2 Carbon metabolism and carbohydrate transporter

*Lactobacillus reuteri* belongs to functional LAB and is able to metabolize carbohydrate to produce lactic acid. The carbon metabolism of *Lactobacillus* include pathway and complex enzyme was important to the utilization of carbohydrates. WHH1689 genome annotation were adopted by GO function categories, and 118 genes involved in carbohydrate transport and metabolism. The 54 genes encoding energy production and conversion were indentified. These transporters consists variety pathway, PEP‐PTS (phosphoenolpyruvate‐phosphotransferase systems), ABC transporter, and Permease. PEP‐PTS transporter was related to the majority of sugars including sucrose, fructose, glucose (Liu et al., 2015; Oberholzer et al., 2005). ABC transporter mainly could make ATP hydrolysis energy with substrate transfer into or out of cells (Roos et al., 2010; Santos et al., 2018). Carbohydrate transporter WHH1689 encodes 15 genes involved in phosphoenolpyruvate synthase and 13 genes for protein phosphotransferase. The 73 genes of ABC transporter in WHH1689 was involved in transporting, including amino acid, ATP‐binding protein, and ABC transporter permease. It is notable that the 29 genes of encoding major facilitator superfamily (MFS) transporter in WHH1689 were indentified. MFS belongs to the secondary transporter family which has effect on physiological processes (Yan, 2013). These results indicate WHH1689 has extensive carbohydrate transporter which could adapt to metabolism pathways.

### 3.3 Carbohydrate‐active enzymes (CAZymes)

Carbohydrate‐Active Enzymes (CAZymes) database was used to analyze genome getting biological information on carbohydrate enzymes. It plays a crucial role in host carbohydrates and intestinal microbe with encoding genes. CAZymes can be found in organisms and nature product, particularly abundant in microorganism (Ardèvol & Rovira, 2015). The CAZymes of WHH1689 contains 21 genes encode glycoside hydrolases (GHs) and 18 genes encode glycosyl transferases (GTs). The 11 genes of WHH1689 were involved in carbohydrate esterases (CEs) and 1genes encoded auxiliary activities (AAs). The 7 genes encoding carbohydrate‐binding modules (CBMs) were indentified (Table 1). Notably, WHH1689 encoded more genes for GHs and GTs which can participate in the metabolism and transport of functional active substances. *Lactobacillus* strains showed functional and structural diversity when carbohydrates were metabolized and transported. GTs and GHs were responsible for their biosynthesis and genetic evolution which also display the carbohydrate binding (Henrissat, Sulzenbacher, & Bourne, 2008).

**Table 1:** Genes related to carbohydrate‐active enzymes of *L. reuteri* WHH1689

Class definition	Gene count	Gene list
Glycoside hydrolases	21	orf00060, orf00264, orf00326, orf00420, orf00968, orf01073, orf01091, orf01185, orf01363, orf01465, orf01497, orf01545, orf01548, orf01565, orf01688, orf01713, orf01939, orf02018, orf01092, orf01088, orf01089
Glycosyl transferases	18	orf00087, orf00088, orf00357, orf00358, orf00688, orf00979, orf00999, orf01145, orf01146, orf01147, orf01324, orf01341, orf01415, orf01416, orf01455, orf01468, orf01479, orf01714
Carbohydrate esterases	11	orf00463, orf00330, orf00880, orf01049, orf01662, orf01698, orf01815, orf01840, orf01856, orf01893, orf01935
Carbohydrate‐binding modules	7	orf00217, orf00479, orf00724, orf01275, orf01386, orf01390, orf01950
Auxiliary activities	1	orf00031

### 3.4 Stress resistance analysis

Lactic acid bacteria are beneficial for human because they have strong tolerance when strains entering gastrointestinal could survive and colonize. *L. reuteri* WHH1689 exhibited higher resistance in simulating gastrointestinal tract and viable counts could be tested after 4 weeks (Chen, Chen, Chen, Ren, Ge, Li, et al., 2018; Chen, Chen, Chen, Ren, Ge, Kang, et al., 2018). Stress regulation mechanism was involved in different aspects, including pH, temperature, osmotic pressure, bile salt, oxidation. The stress‐related proteins not only can reveal genetic adaptation but also regulate evolution resistance (Boden & Merali, 2001). Analysis of stress‐related proteins of *L. reuteri* WHH1689 was shown in Table 2. WHH1689 contains two genes related to alkaline phosphatase and two genes encode alkaline shock protein Asp23 which is linked to cell membrane improving Gram‐positive bacteria tolerance. Moreover, five genes encode sodium‐proton antiporter which evaluated the energy of the Na^+^ and H^+^ movement for converting the function of transporters, five genes encoded F0F1 ATP synthase which was related with ATP synthesis utilizing ion translocation (Zhang et al., 2017). The potential functional information was obtained from genome of WHH1689. Tolerance test was necessary to verify the functionality of the strain. We studied the stress resistance of *L. reuteri* WHH1689 which showed stable vitality to different treatment (Table 3). With exposure to pH 2–10, the maximum survival rate occurred at pH 3 of 89.16% ± 0.23. Under acidic and alkaline conditions, this strain could show high activity of bacteria, it still displayed survival of 42.86% ± 0.19 when incubated in extreme pH 10. The genetic component indicated that the F0F1 ATP synthase, alkaline phosphatase, and shock protein of *L. reuteri* WHH1689 reveal it may function in ATP‐dependent proton and adapt in alkaline and acid environment.

**Table 2:** Stress‐related proteins of *L. reuteri* WHH1689

Stress factors	Related proteins	Locus tag
pH	Alkaline shock protein	orf1237, orf00906
	Alkaline phosphatase	orf01996, orf01889
	Sodium‐proton antiporter	orf00503, orf00504, orf00183, orf01837, orf01990,
	F0F1 ATP synthase	orf00536, orf00537, orf00538, orf00539, orf00541,
Bile	Choloylglycine hydrolase	orf00832
	Inorganic pyrophosphatase	orf00901
Temperature	Cold shock protein CspA	orf00697, orf01643
	HrcA family transcriptional regulator	orf00804
	Heat shock protein GrpE	orf00805
	Heat shock protein DnaK	orf00806
	Heat shock protein DnaJ	orf00807
	Heat shock protein Hsp33	orf00300
	Heat shock protein Hsp20	orf01381
	Molecular chaperone GroES	orf00399
	Molecular chaperone GroEL	orf00400
	Heat shock protein HtpX	orf00270
Osmotic pressure	Choline	orf01452
Oxidation	Glutathione reductase	orf01513
	NADH oxidase	orf00076, orf01790
	NADH‐dependent flavin reductase	orf00102
	NADH‐dependent oxidoreductase	orf00146
	NADH dehydrogenase	orf00594
	NADH‐flavin reductase	orf00178

**Table 3:** Survival (%, mean ± *SD*) of tolerance assay by *L. reuteri* WHH1689

Treatment	Survival rate (%)
Control	100.8 ± 0.19
pH	
2.0, 3 hr	71.27 ± 0.07
3.0, 3 hr	89.16 ± 0.23
4.0, 3 hr	85.32 ± 0.17
5.0, 3 hr	83.26 ± 0.12
6.0, 3 hr	78.32 ± 0.19
7.0, 3 hr	75.66 ± 0.21
8.0, 3 hr	70.23 ± 0.19
9.0, 3 hr	50.35 ± 0.11
10.0, 3 hr	42.86 ± 0.19
Bile salt	
0.2%, 3 hr	99.86 ± 0.13
0.3%, 3 hr	96.09 ± 0.21
0.4%, 3 hr	95.66 ± 0.17
0.5%, 3 hr	92.30 ± 0.19
Sodium chloride	
6.0%, 24 hr	86.82 ± 0.11
7.0%, 24 hr	85.66 ± 0.21
8.0%, 24 hr	78.88 ± 0.05

Lactic acid bacteria has tolerance ability to produce bile salt hydrolases (BSH) induced micelles subject to conjugated bile salts (CBAs). Two genes encoded that choloylglycine hydrolase and inorganic pyrophosphatase which were involved in CBAs. In this paper, effect of bile salts on the strains survival demonstrated WHH1689 could tolerate to gastrointestinal environment. With exposure to 0.2%, 0.3%, 0.4%, 0.5% bile salts, the survival rate was almost more than 90%. The genes of WHH1689 encoded inorganic pyrophosphatase may maintain surface tension of membrane and keep membrane integrity to improve strain tolerance.

Furthermore, the WHH1689 genome encoded one choline protein which can regulate the osmotic pressure of the cell membrane. High salinity caused sensitive membrane to dehydrate influence osmolality of bacteria. Table 3 shows tolerance of WHH1689 to sodium chloride. With exposure to 6.0%, 7.0%, 8.0% sodium chloride solution, the strain maintained stable survival rate. With the increase of salt concentration, the survival of WHH1689 gradually decreased. It exhibited survival of 78.88% ± 0.05 when incubated in 8.0% salt solution. The tolerance of osmotic pressure revealed it could potentially apply in kimchi and another salted food.

**Figure 4:**
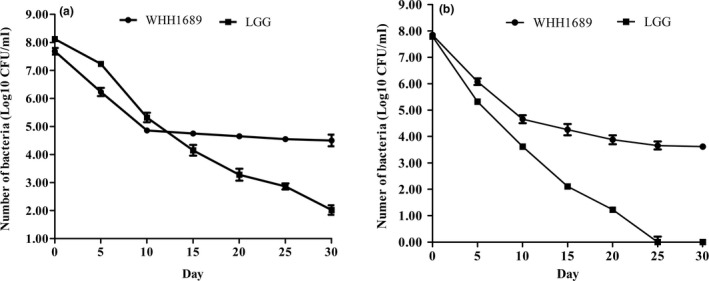
Viability counts of probiotics (*Lactobacillus reuteri* WHH1689, ●), *Lactobacillus rhamnosus* GG, ▲) stored at 45°C (a) and 50°C (b) over 30 days

WHH1689 encodes most genes related to temperature stress comparing to the gene number of BSH. Ten genes were detected in WHH1689 including cold and heat shock protein. Cold shock CspA protein has effect on nucleic acid‐binding to serve as transcriptional regulators. A cluster of heat shock protein can be found: hrcA‐grpE‐dnaK‐dnaJ. HcA protein was involved in DNA binding and GrpE was regarded as a thermosensor of the DnaK system. DnaJ, GrpE represented response changes to adapt environment temperature (Susin, Baldini, Gueiros‐Filho, & Gomes, 2006). GrpE‐dnaK‐dnaJ may express resistance to stress under acid conditions in *Acetobacter pasteurianus* NBRC 3283 (Ishikawa et al., 2010). In previous research, *L. reuteri* WHH1689 could survive at 28 and 37°C for 4 weeks (Chen, Chen, Chen, Ren, Ge, Li, et al., 2018; Chen, Chen, Chen, Ren, Ge, Kang, et al., 2018). The genetic information revealed heat shock genes were more than cold shock gene which indirectly illustrated that this strain had tolerance at relatively high temperature. *L. reuteri* WHH1689 was incorporated into MRS broth at 45 and 50°C for 30 days. LGG was used as control strain. Figure 4 displayed the viable bacteria of the strains stored at 45 and 50°C. At 45°C, the viable counts of WHH1689 decreased 3 log during 30 days of storage, whereas the counts of LGG decreased significantly for 6 log (Figure 4a). At 50°C, the viable counts of WHH1689 were detected to be about 4.0 log after 30 days and the counts of LGG were not determined after 25 days (Figure 4b). The gene analysis had been consistent with the results, which showed that WHH1689 may be a potential strain that can survive in high temperature.

**Figure 5:**
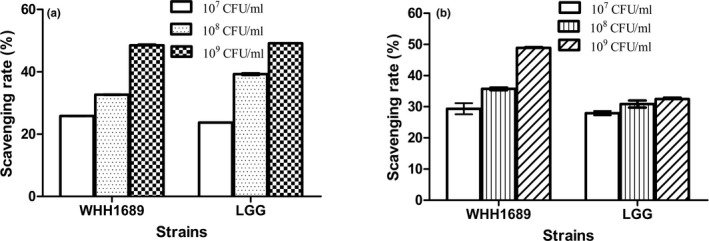
Scavenging activities on hydroxyl radicals using different concentrations of *L. reuteri* WHH1689, *reuteri* WHH1689, and *L. rhamnosus* GG (a). Scavenging of DPPH radicals by *L. reuteri* WHH1689 and *L. rhamnosus* GG (b)

Oxidative stress refers to the essential balance in aerobic metabolism. Seven genes encoded oxidase, reductase, and dehydrogenase were identified which may have potential oxidation resistance (Sachan, Johnsen, & Hongu, 2012). We studied the scavenging for hydroxyl radical and DPPH radical of strains. Figure 5a showed scavenging for hydroxyl radical of LGG and WHH1689. *L. rhamnosus* GG (LGG) proved to have significant effect on antioxidants and superoxide dismutase which was used as for contrast strain (Goyal, Rishi, & Shukla, 2013). Both the strains demonstrated scavenging activity of hydroxyl radical in the concentration range of 10^7^–10^9 ^CFU/ml. With the counts of bacteria increased, the scavenging rate showed an upward trend. The maximum rate was occurred at the concentration range of 10^9 ^CFU/ml. The strains WHH1689 and LGG, respectively, had scavenging of 48.68% ± 0.16 and 49.06% ± 0.09. The results were not significant between two strains indicated WHH1689 was capable for scavenging hydroxyl radical.

For DPPH radical in Figure 5b, as the concentration changes, scavenging rate gradually increased for WHH1689; however, there was no significant changes in different concentration for LGG. The scavenging reached the highest value when the concentration of counts was 10^9 ^CFU/ml. The WHH1689 strain had a scavenging of 49.28% ± 0.18, whereas strain LGG had a scavenging of 32.15%. These researches demonstrated WHH1689 may be an antioxidant probiotic. Genome analysis and functional experiment indicated that *L. reuteri* WHH1689 could have antioxidant ability for genetic adaptions.

### 3.5 Adhesion ability

The adhesion of LAB indicated that cells can adhere to small intestinal epithelial cell surface, and the mechanism was related to hydrophobicity and potentially surface exposed (PSE) proteins. PSE protein play crucial role in adhesion or binding to cell surface (Barinov et al., 2009). The genome analysis showed that WHH1689 contained gene encode fibronectin‐binding protein (orf00991), lipoprotein signal peptidase (orf00987, orf01257), maltose phosphorylase (orf00060), triosephosphate isomerase (TPI, orf00265, orf00451, orf00189). TPI was associated with glycolysis which can be released to organism for acclimatization and improve adhesion ability (Helfert, Estévez, Bakker, Michels, & Clayton, 2001) which may deliver benefit effect for health.

We examined the adhesion ability of WHH1689 and LGG at different concentrations to HT‐29 epithelial cells in Table 4. From 10^6^ to 10^9 ^CFU/ml, both of the strains showed high adhesion rate. The WHH1689 still had an adhesion of 4.02% ± 1.16 when the viable of counts was at lower concentration of 10^6 ^CFU/ml. The adhesion of WHH1689 was almost consistent with LGG. Adhesive ability of this strain to HT‐29 epithelial cells indicated it may preferably function in intestinal tract.

**Table 4:** Adherence ability (%, mean ± *SD*)

Concentration	Adherence rate (%)	
	*Lactobacillus reuteri* WHH1689	*Lactobacillus rhamnosus* GG
10^6^ CFU/ml	4.02 ± 1.16	4.16 ± 1.18
10^7^ CFU/ml	4.35 ± 1.08	4.28 ± 1.16
10^8^ CFU/ml	4.12 ± 1.68	4.61 ± 1.65
10^9^ CFU/ml	4.26 ± 1.05	4.28 ± 1.22

### 3.6 Folic acid biosynthesis genes

**Figure 6:**
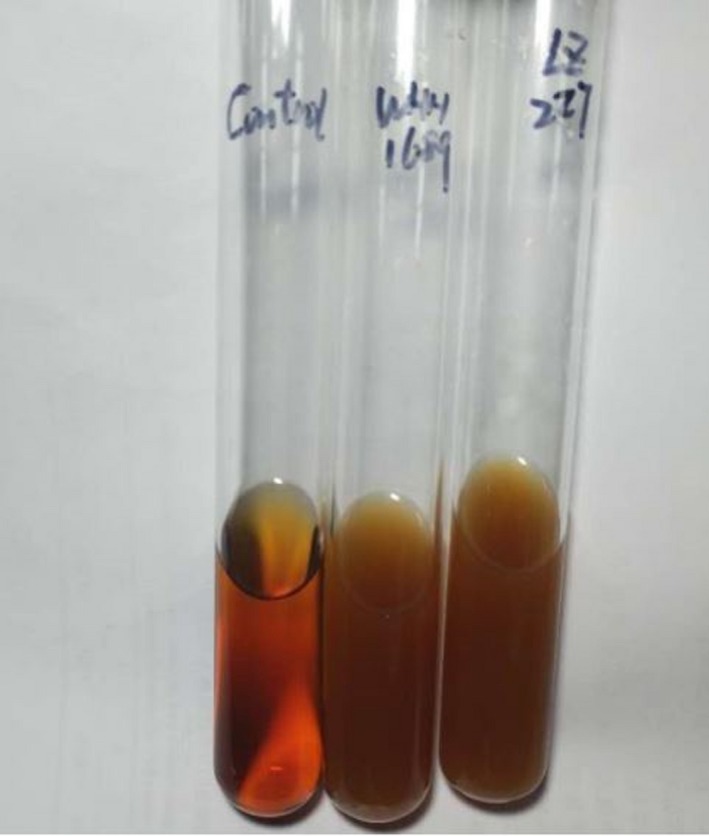
Folate production of *L. reuteri* WHH1689

Folic acid is commonly found in all kinds of food such as plant, vegetable, fruit, and meat food which is an important substance involved in nucleic acid synthesis and cell differentiation. The most important physiological function of folic acid is the influence of deficiency on the development of fetal nervous system. The majority of LAB was folic acid deficient strains, however, some have the ability to synthesize it. A series of enzymes catalyzed guanine nucleoside triphosphate (GTP) production by purine metabolism could form folic acid (Bolin & Cardozo‐Pelaez, 2007). In WHH1689 genome, we found that enzymes related to GTP pathway, folA (orf00877), folB (orf01353), folC (orf01350, orf00595), folD (orf01235), folE (orf01351), folk (orf01352), folP (orf01348), suggesting potential folic acid biosynthesis of the strain (Licciardi, Tang, Billingham, Armes, & Lewis, 2005). We tested the ability of folic acid production by *L. reuteri* WHH1689. *L. plantarum* LZ227 has been reported as probiotic strain producing B‐group vitamins used as the comparative strain in Figure 6 (Li, Zhou, & Gu, 2016). Both of the two strains grew well on folic acid assay medium indicated WHH1689 could produce folate. The folic acid content of cell supernatants produced by WHH1689 was 476.0 μg/L, and intracellular extracts had a folate content of 27.5 μg/L.

### 3.7 Comparative genomic analysis of special genes

We selected *L. reuteri* DSM 20016, *L. reuteri* TD1, *L. reuteri* SD2112, *L. reuteri* I5007 for comparative genomic, which have been whole genome‐sequenced and deposited in GenBank. The amount and annotation of special genes have been showed base on comparative genome (Table 5). The data indicated that WHH1689 existed special genes after comparison with other strains, 16 genes encoded IS*30* family, and 15 genes encoded IS*3* transposase. Another hypothetical protein and transposase have not completely annotated. It was noteworthy that more genes encoded ISL*3* family transposase and IS*4* transposase in WHH1689. Insertion sequence (IS) distributed in various bacteria are transferring genetic components which could have multiple effects on information transfer and extreme adaptation. They are capable of independent transposition and improving genetic variation (Szabó, Kiss, & Olasz, 2010). The IS*30* family present in most bacteria including Gram‐positive and Gram‐negative bacteria and they could be distributed in various *Lactobacillus* spp (Kumar, Grover, Kaushik, & Batish, 2014). Furthermore, IS*30* elements have been proved to be associated with environmental adaption and stress resistance, due to hypothetical genes involved in carbohydrate metabolism which was benefit for gastrointestinal microflora colonization (El et al., 2012). Special genes of WHH1689 coded IS*3* transposase have been involved in host adaptation and contribution for genetic diversity. IS*4* transposase conserved within its family display similar function with IS*3* families. ISL*3* element was discovered in *Lactobacillus delbrueckii* subsp which conjugative with two genes play a significance role in milk fermentation (Lysnyansky et al., 2009). Transposition of ISL*3* could potentially influence on the expression of adjacent genes (Morel et al., 2017). Therefore, specific genes mostly related to the ISs family speculated WHH1689 could be of benefit for genetic adaptation and stress resistance.

**Table 5:** Special genes of comparative genome

WHH1689	DSM20016	I5007	SD2112	TD1	COG annotation
16	0	0	0	0	IS30 family
15	0	0	0	0	IS3 transposase
14	1	3	1	5	Transposase
13	1	1	0	5	Hypothetical protein
11	0	1	0	0	Hypothetical protein
11	0	1	0	0	Possible integrase, partial
11	0	0	0	0	Hypothetical protein
10	0	0	0	0	Transposase
24	1	15	2	1	IS6501 element
23	4	9	11	5	Hypothetical protein
20	11	2	25	17	RNA‐directed DNA polymerase
20	1	4	15	0	Hypothetical protein
18	5	17	18	5	Hypothetical protein
9	1	1	10	0	ISL3 element
7	1	6	3	1	Transposase
6	2	5	6	0	Integrase
6	2	3	4	1	IS4 transposase
6	1	0	0	0	Transposase

### 3.8 Diversity of adaptation

**Figure 7:**
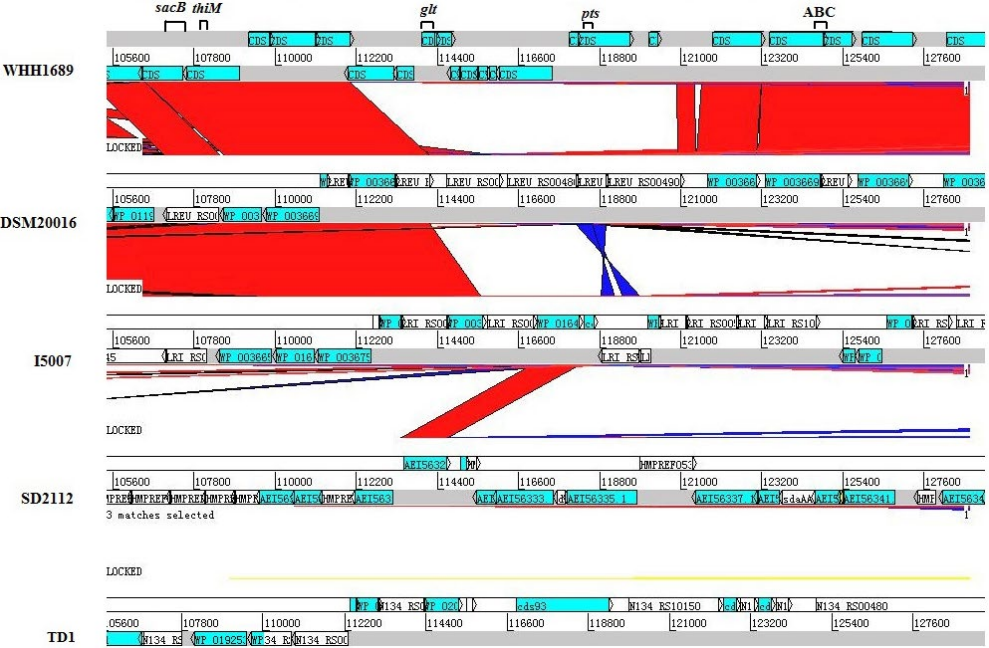
Diversity of adaptation region genes in *L. reuteri* strains. Functional gene of adaptation diversity based on comparative genome. The red and blue region illustrated high sequence identity and reverse direction. The genes including fructosyltransferase (*sacB*), hydroxyethylthiazole kinase (*thiM*), glycosyl transferase family (*glt*), PTS sugar transporter (*pts*), and methionine ABC transporter (ABC)

Comparative genome of *L. reuteri* display diversity of lifestyle in cellular component, molecular function, and biological progress. The 285 kb region from 105,600 to 125,400 exclusively encodes special genes of WHH1689. The region is much longer than other *L. reuteri* strains (Figure 7). Moreover, this whole region has the higher GC content (68.7%), indicating that most genes have been involved in genetic evolution. The special cluster, including fructosyltransferase (*sacB*), hydroxyethylthiazole kinase (*thiM*), glycosyl transferase family (*glt*), PTS sugar transporter (*pts*), and methionine ABC transporter (ABC), are indicated in WHH1689. Fructosyltransferase (*sacB*) is available in carbohydrate transport, which possess biochemical function that could play crucial roles in stress tolerance (Porrasdomínguez, ÁvilaFernández, Mirandamolina, Rodríguezalegría, & Munguía, 2015). Hydroxyethylthiazole kinase (*thiM*) is an essential enzyme which effect on the metabolism of vitamin B1. Glycosyltransferases regulated the glycan expression and adjusted molecular mechanism, contribution for carbohydrate metabolism (Furukawa, Takamiya, Okada, Inoue, & Fukumoto, 2001; Mckinnell, Bartsch, Lee, Huang, & Miller, 2014). PTS and ABC transporter contains *lacS*,* galP*,* rafP*, and *metQ* could provide various transport pathways and improve adaptation (Gunnewijk & Poolman, 2000; Hollenstein, Frei, & Locher, 2007). This special region reveals WHH1689 could be capable for stress resistance and adapt effectively to different environmental conditions.

## 4 CONCLUSIONS

*Lactobacillus reuteri* WHH1689 isolated from Chinese traditional which has high viability and low postacidification in a room‐temperature‐storage drinkable. In this paper, we revealed genes related to carbon metabolism pathway, folic acid biosynthesis, stress resistance and adaptation diversity based on comparative genomic analysis. Functional assay confirmed gene prediction identified probiotic properties of WHH1689. These results could provide genetic basis for long‐term survival and probiotic function, especially in environment adaptation. Further work we will be needed to research on transcriptome and metabonomics. Transcriptome profiling analysis may reveal metabolic changes across various growth conditions in *Lactobacillus* strains.

## CONFLICT OF INTEREST

The authors have no conflict of interests to declare.

## ETHICAL STATEMENT

This study does not involve any human or animal testing.
